# Drug-induced sleep endoscopy (DISE) for non-CPAP treatment selection in patients with sleep-disordered breathing

**DOI:** 10.1007/s11325-012-0671-9

**Published:** 2012-02-26

**Authors:** Olivier M. Vanderveken

**Affiliations:** 1 Faculty of Medicine and Health Sciences, University of Antwerp, Antwerp, Belgium; 2 Department of Otorhinolaryngology-Head and Neck Surgery, and, Sleep Disorders Center, University of Antwerp, Antwerp University Hospital, Wilrijkstraat 10, 2650 Edegem, Antwerp Belgium

The aim of upper airway (UA) evaluation in patients with sleep-disordered breathing (SDB) is not only to gain a better insight into the complex pathophysiology of UA collapse but also to improve treatment success rates while prospectively selecting the most appropriate therapeutic option for the individual patient [1]. UA assessment in SDB patients is often limited by the fact that the evaluation is static and/or performed during wakefulness, possibly not representing the actual dynamics of UA collapsibility during sleep, while investigations of the UA during natural sleep remain time and manpower consuming [1–3]. Drug-induced sleep endoscopy (DISE) provides an alternative method of studying the UA while performing a fiberoptic endoscopy during sedation as pharmacologically induced with midazolam and/or propofol [1–4]. DISE allows to determine the pattern of UA narrowing and obstruction, as part of the therapeutic decision-making process towards UA surgery and/or oral appliance therapy in patients with SDB as there is a high interest in the prospective prediction of non-CPAP treatment outcome [2, 4, 5]. Recent studies have examined the validity as well as the test–retest and interrater reliability of DISE [6–8]. DISE procedures, however, lack uniformity in the methods used for the drug-induced sedation, while a consensus on DISE classification systems has not been established [3–5, 9].

In this issue of *Sleep and Breathing*, Eichler and colleagues compared the treatment recommendations that would be given after a clinical basic ENT examination (CBE) with the recommended therapy after conducting a DISE [10]. The results of the reported study indicate that DISE had a relevant impact on the treatment recommendation, and, thus, could possibly change the success rates of non-CPAP therapy in SDB patients [10].

Mandibular advancement device (MAD) treatment represents the main non-CPAP therapy for patients with SDB [11, 12]. A custom-made, titratable MAD has been recommended [13–15]. Currently, there is no reliable way to prospectively predict the outcome of MAD treatment in the individual patient [11]. As in previous reports, Eichler et al. have been using a simultaneous mandibular protrusion maneuver during DISE to assess whether mandibular advancement leads to a visible enhancement of the UA dimensions, as a possible predictor of successful MAD treatment [3–5, 10]. In the reported study, a modified jaw thrust maneuver (Esmarch) was performed, by closing the mouth and lightly pushing forward the mandible on both sides [10]. This approach has the potential disadvantage of not adequately accounting for the given thickness of a particular MAD, whereas each oral appliance inherently causes a certain amount of vertical mouth opening [16]. In addition, similar to the mandibular protrusion maneuver as reported by Johal et al., the Esmarch maneuver is not reproducible in terms of the degree of mandibular advancement [3, 5, 10]. Recently, a novel approach using a custom-made simulation bite in maximal comfortable protrusion during DISE has been described for the prediction of the outcome with MAD treatment [4]. This technique might at least offer the advantage of performing a reproducible mandibular maneuver, also accounting for the given thickness of a MAD, but does not take into account the titratable aspect of the currently recommended titratable MAD treatment neither.

Sleep surgery procedures are directed at specific collapsible UA structures and, therefore, DISE may add to a proper selection of a specific surgical procedure for an individual patient [2, 10]. In the reported study the most frequent site of UA obstruction visualized by DISE was the palatal level, which is indeed in line with other recent reports [3]. For all treatment sites, except for the treatment of the tonsils, a statistically significant disagreement was noted as recommended by CBE versus DISE. Interestingly, the treatment recommendations towards tongue base interventions and MAD treatment had the highest range of change based on DISE as compared to CBE [10].

Eichler and colleagues have to be congratulated for this well-designed study with two different ENT specialists performing the CBE versus the DISE independently from each other, while a third ENT specialist compared CBE with DISE findings and recommendations towards the type of non-CPAP treatment. The detailed description of the clinical criteria used for the treatment recommendations based on CBE and DISE certainly adds to the quality of this publication by Eichler et al. [10]. Based on the results of the reported study, an improvement in success rates of non-CPAP therapies for SDB due to DISE can be assumed. The results of this study might suggest that DISE is most relevant when considering tongue base surgery or MAD therapy; and that DISE might turn out to provide less added value for the identification of indications for the treatment of the tonsils [10]. As discussed previously, a possible criticism of the reported study is that a non-reproducible and non-titratable mandibular protrusion maneuver has been performed during DISE, also not accounting for vertical opening while closing the mouth.

DISE is increasingly performed offering the possibility of dynamic UA evaluation during artificial sleep as a promising technique to select the proper non-CPAP treatment for patients with SDB. On the other hand, DISE has potential limitations as a patient’s selection tool. First, DISE lacks a uniform method of sedation (bolus versus target controlled infusion; midazolam and/or propofol administration) [3–5, 10]. Secondly, many different DISE classification systems have been introduced in literature, without consensus [3–5, 9]. Finally, the mandibular advancement maneuvers performed during DISE lack reproducibility and standardization up to this date. As a result, the future challenges, among other research topics, are to outline a standardized method of sedation, to identify a uniform DISE classification system, and, to define a reproducible and titratable standard mandibular advancement maneuver during DISE.
